# 

**Published:** 1913-04

**Authors:** 


### CONTENTS.

ORIGINAL COMMUNICATIONS:— Page

Syphilis from the Standpoints of the Syphilitic and the General Public. By Cosby Swanson, M. D., Atlanta, Ga. .......... 1

Clinical Society of the New York Polyclinic Medical School and Hospital. By Dr. Anthony Bassler ... 19

EDITORIAL .......... 23

SELECTIONS AND ABSTRACTS .......... 26

NEWS AND NOTES .......... 43

BOOK REVIEWS .......... 45

